# Ten simple rules for getting started with command-line bioinformatics

**DOI:** 10.1371/journal.pcbi.1008645

**Published:** 2021-02-18

**Authors:** Parice A. Brandies, Carolyn J. Hogg

**Affiliations:** School of Life and Environmental Sciences, Faculty of Science, The University of Sydney, Sydney, New South Wales, Australia; Dassault Systemes BIOVIA, UNITED STATES

## Introduction

**Fig 1:**
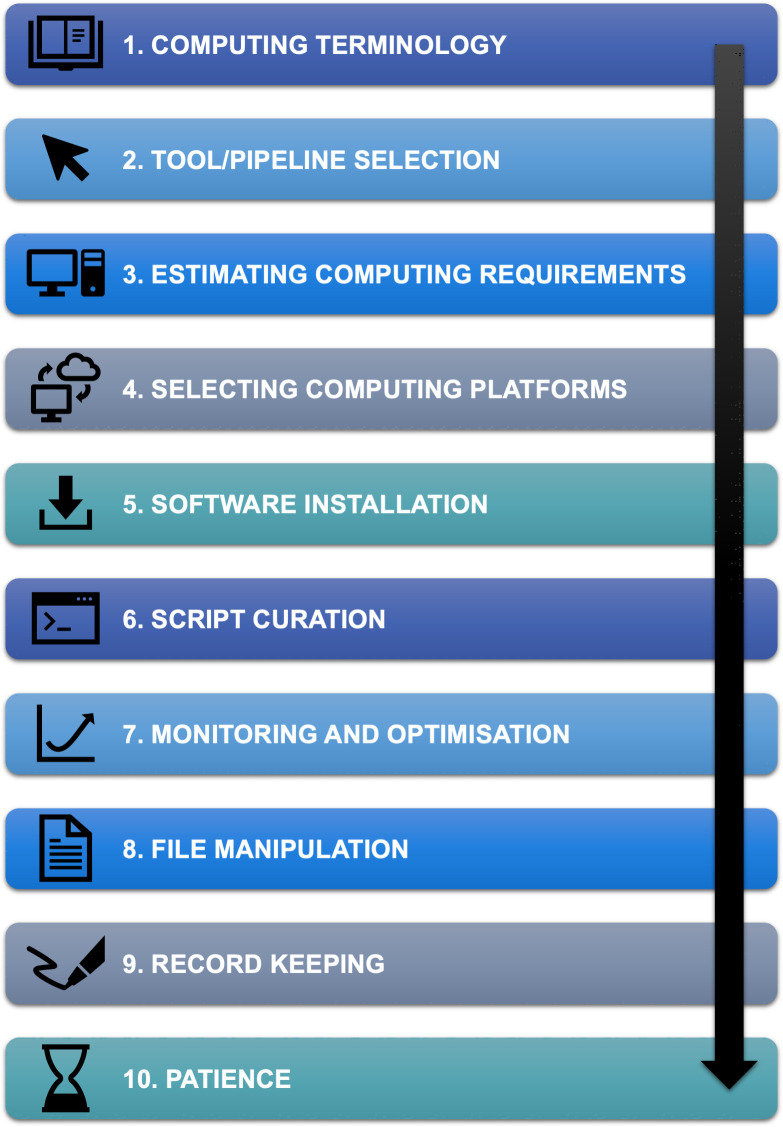
Our 10-step process for getting started with command-line bioinformatics. Each step corresponds to each of our 10 simple rules presented below.

Sequencing technologies are becoming more advanced and affordable than ever before. In response, growing international consortia such as the Earth BioGenomes Project (EBP) [1], the Genome 10K project (G10K) [2,3], the Global Invertebrate Genomics Alliance (GIGA) [4,5], the Insect 5K project (i5K) [6,7], the 10,000 plants project (10KP) [8], and many others have big plans to sequence all life on earth. These consortia aim to utilise genomic data to uncover the biological secrets of our planet’s biodiversity and apply this knowledge to real-world matters, such as improving our understanding of species’ evolution, assisting with conservation of threatened species, and identifying new targets for medical, agricultural, or industrial purposes [1]. All of these goals rely on someone to analyse and make sense of the tremendous amounts of biological data, making bioinformaticians more sought-after than ever. Many researchers with a background in biology and genetics are stepping up to the challenge of big data analysis, but it can be a little daunting to start down the path of bioinformatics, particularly using the command line, without a strong background in computing and/or computer science. A recent “Ten simple rules” article highlighted the importance of bioinformatics research support [9]. Here we provide 10 simple rules for anyone interested in taking the leap into the realm of bioinformatics using the command line. We have put together these 10 simple rules for those starting on their bioinformatics journey, whether you be a student, an experienced biologist or geneticist, or anyone else who may be interested in this emerging field. The rules are presented in chronological order, together encompassing a simple 10-step process for getting started with command-line bioinformatics (Fig 1). This is by no means an exhaustive introduction to bioinformatics, but rather a simple guide to the key components to get you started on your way to unlocking the true potential of biological big data.

### Rule 1: Get familiar with computer terminology

The first step in your command-line bioinformatics journey can be overwhelming due to the wealth of new terminology. This is where you need to channel your inner computer geek and learn the new language of computer terminology. In fact, this very paper is riddled with it, so our first rule addresses this tricky obstacle. Having a basic understanding of computing and associated terminology can be really useful in determining how to run your bioinformatics pipelines effectively. It can also help you troubleshoot many errors along the way. Understanding the terminology allows you to talk with your institutional information technology (IT) departments and communicate your computational needs to answer your biological questions. This will allow you to be able to source the resources you will need. A number of basic definitions of the main terms that you will likely come across as you enter the world of bioinformatics is presented in Box 1.

**Box 1. Some simple definitions of common computer terms**

Algorithm: The set of rules or calculations that are performed by a computer program. Certain algorithms may be more suitable for particular datasets and may have differences in performance (e.g., in speed or accuracy).

Central processing unit (CPU): The chip that performs the actual computation on a compute node or VM.

Compute node: An individual computer that contains a number of CPUs and associated RAM.

Core: Part of a CPU. Single-core processors contain 1 core per CPU, meaning CPUs and cores are often interchangeable terms.

CPU time: The time CPUs have spent actually processing data (often CPU time ~ = Walltime * Number of CPUs).

Dependency: Software that is required by another tool or pipeline for successful execution.

Executable: The file that contains a tool/program. Some software has a single executable, while others have multiple executables for different commands/steps.

High performance computer (HPC): A collection of connected compute nodes.

Operating system (OS): The base software that supports a computer's basic functions. Some of the most common linux-based operating systems include those of the Debian distribution (Ubuntu) and those of the RedHat distribution (Fedora and CentOS).

Pipeline: A pipeline is a workflow consisting of a variety of steps (commands) and/or tools that process a given set of inputs to create the desired output files.

Programming languages: Specific syntax and rules for instructing a computer to perform specific tasks. Common programming language used in bioinformatics include Bash, Python, Perl, R, C, and C++.

Random access memory (RAM): Temporarily stores all the information the CPUs require (can be accessed by all of the CPUs on the associated node or VM).

Scheduler: Manages jobs (scripts) running on shared HPC environments. Some common schedulers include SLURM, PBS, Torque, and SGE.

Script: A file which contains code to be executed in a single programming language.

Thread: Number of computations that a program can perform concurrently—depends on the number of cores (usually 1 core = 1 thread).

Tool: A software program that performs an analysis on an input dataset to extract meaningful outputs/information—Tool, software, and program are often used interchangeably but refer to the core components of bioinformatics pipelines.

VM: Virtual machine—Similar to a compute node as it behaves as a single computer and contains a desired number of CPUs and associated RAM (usually associated with cloud computing).

Walltime: The time a program takes to run in our clock-on-the-wall time.

### Rule 2: Know your data and needs to determine which tool or pipeline to use

This can often be one of the most difficult steps as there are usually many different tools and pipelines to choose from for each particular bioinformatic analysis. While you may think about creating your own tool to perform a particular task, more often than not, there is already a preexisting tool that will suit your needs, or perhaps only need minor tweaking to achieve the required result. Having a clear understanding of your data and the types of questions you are wanting to ask will go a long way to assisting in your tool or pipeline selection. Selecting the most suitable pipeline or tool will be dependent on a number of factors including:

#### Your target species and quality of data

Some bioinformatic pipelines/software may work better for a particular species based on their unique features (e.g., genome size, repeat complexity, ploidy, etc.) or based on the quality of data (e.g., scaffold length, short reads versus long reads, etc.). Reading other published papers on similar species will assist with being able to define this.

#### Your available computing resources and time restrictions

Certain software may be based of different algorithms which can result in significant reductions or increases of computational resources and walltime. Some shared HPC infrastructure may have walltime limitations in place, or the amount of RAM or cores may be a limiting factor when using personal computing resources. Make enquiries with your institutional IT department regarding limits on personal computing or HPC infrastructure before you start.

#### Which tools are readily available

Many bioinformatic pipelines and tools are freely available for researchers, though some require purchasing of a license. Additionally, some tools/pipelines may already be available on your desired computing infrastructure or through your local institution. There are a number of “standard” bioinformatic command line tools that have broad applicability across a variety of genomic contexts and are therefore likely already installed on shared infrastructure. Such examples include tabix, FastQC, samtools, vcftools/bcftools, bedtools, GATK, BWA, PLINK, and BUSCO. Furthermore, collaborators or other researchers may have already tested and optimised a particular pipeline on a certain infrastructure and have therefore already overcome the first hurdle for you.

Talking with colleagues who are working on similar projects and reading through the literature is often the best way to decide on which software to use for a particular analysis. There are many publications that benchmark different tools and compare the advantages and disadvantages of similar pipelines. There are also many online web forums (e.g., BioStars [10]) that may also assist with your decision-making process. Be sure to search through the different web forums to see whether another researcher has also asked the same or similar question as you (this is often the case). If you cannot find a solution, ensure any questions you post are clear and detailed, with examples of code or errors provided to have the best chance of helpful replies and answers. Beginning with a pipeline that has previously been tested and optimised on a particular platform is helpful in getting a head start, though do not be scared to try out a new or different pipeline if it seems better suited to your data or desired outcome.

### Rule 3: Estimate your computing requirements

Once you have selected your desired tool or pipeline, the next crucial step involves estimating the desired computing requirements for your chosen analysis. Estimating your requirements will not only allow you to determine which platforms may be most suitable to run your pipeline (e.g., cloud versus HPC; see Rule 4) but will also reduce time spent on troubleshooting basic resource errors (e.g., running out of RAM or storage space). Furthermore, this step is almost always necessary prior to running any tool or pipeline on any given compute infrastructure. For instance, on shared HPC environments, your job script will need to include your requested computational resources (cores, RAM, walltime), and you will need to make sure you have enough disk space available for your account. Similarly, for cloud computing, you will need to decide what size machine/s (cores and RAM) and how much attached storage you need for your analysis. Estimating incorrectly can be frustrating as you will waste time in queues on shared HPC infrastructure, only to have your analysis terminated prematurely, or waste money in the cloud specifying more resources than you actually need. Many bioinformatics tools can be run on a single core by default, but this can result in much greater walltimes [11] (which are often restricted on shared HPC infrastructure). Increasing the number of cores can greatly reduce your walltime, though there is often a balance between this and other important factors such as RAM usage, cost, queueing time, etc. [11].

It can be a little tricky estimating computing requirements for a pipeline you have never run before, or on a species that the pipeline has never been tested with before. Never fear though as there are a number of places you can seek out information on computing requirements. First and foremost, read the documentation for the pipeline/tool you are running. Some tool documentation will provide an example of the compute resources required or provide suggestions. Additionally, many programs will provide a test dataset to ensure the pipeline is working correctly before employing your own datasets. These test datasets are a great start for estimating minimal computational requirements and to obtain some general benchmarks when using different parameters or computing resources. If the tool documentation does not provide a guide of computing requirements or an example dataset, you may wish to use a smaller subset of your own data for initial testing. The literature may also provide a guide for general computing requirements that have been used for a particular tool or pipeline for a similar species or sample size. There are many publications where common bioinformatics pipelines are compared with one another to assess performance and results across a variety of organisms (e.g., [12–15]). These can be found with a simple citation search. Finally, another great resource for estimating your computing requirements is from other researchers. Talking to others in your field who may work with similar data or utilising online forums such as BioStars [10] will assist in understanding the resources required.

In general, 32 cores and 128 GB of RAM is usually sufficient for most common bioinformatics pipelines to run within a reasonable timeframe. With that being said, some programs might require much less than this, while others may have much higher memory requirements or enable greater parallelisation.

### Rule 4: Explore different computing options

After estimating your computing requirements for your chosen pipeline, you will then need to determine where such resources are available and which infrastructure will best suit your needs. Some tools may easily run on a personal computer, though many of the large bioinformatics pipelines (particularly when working on organisms with large genomes like mammals and plants) require computational resources that will well exceed a standard PC. Many institutions have a local HPC or access to national/international HPC infrastructure. However, the unprecedented generation of sequencing data has started to push these shared infrastructures to their limits. These resources are not always well suited to the requirements of bioinformatic pipelines such as their high I/O demands and “bursty” nature (see Rule 7) [16]. This is why cloud computing is becoming increasingly popular for bioinformaticians [16–20].

Cloud computing provides a number of key advantages over traditional shared HPC resources including:

- The ability to tailor your computing resources for each bioinformatic tool or pipeline you wish to use;
- Complete control over your computing environment (i.e., operating system, software installation, file system structure, etc.);
- Absence of a queuing system resulting in faster time to research;
- Unlimited scalability and ease of reproducibility.

Utilising cloud resources also prevents the need for researchers to purchase and maintain their own physical computer hardware (which can be time consuming, costly, and nowhere near as scalable [21]). However, commercial cloud computing does come at a cost and can be a bit of a steep learning curve. Fortunately, services like RONIN (https://ronin.cloud) have simplified the use of cloud computing for researchers and allow for simple budgeting and cost monitoring to ensure research can be conducted in a simple, cost-effective manner. Researchers at academic institutions may also have access to other free cloud compute services such as Galaxy (https://usegalaxy.org/), ecocloud (https://ecocloud.org.au/), nectar (https://nectar.org.au/cloudpage/), and CyVerse (https://www.cyverse.org).

Overall, deciding where to run your analysis will be dependent on your data/species, what platforms are most easily accessible to you, your prior experience, your timeline, and your budget. Exploring different compute options will allow you to choose which infrastructure best suits your needs and enable you to adapt to the fast-evolving world of bioinformatics.

### Rule 5: Understand the basics of software installation

When wanting to utilise a personal resource for your bioinformatic pipelines, such as a cloud VM or a personal computer, you will need to get familiar with the various installation methods for your required tools. While software installation is sometimes provided as a service for some shared HPC platforms, understanding the basics of software installation is useful in helping you troubleshoot any installation-based errors and identify which software you can likely install locally yourself (i.e., without requiring root user privileges). There are numerous ways software can be installed, but we have provided 4 main methods that should cover most bioinformatics software (Box 2).

**Box 2. Common software installation methods for bioinformatics tools**

**Package managers**

APT (Advanced Package Tool) (https://www.debian.org/doc/manuals/apt-guide/index.en.html) is a package manager that is often already installed by default on many Debian distributions and enables very simple installation of available tools. APT works with a variety of core libraries to automate the download, configuration, and installation of software packages and their dependencies. A number of common bioinformatics tools are available through APT including NCBI blast+, samtools, hmmer, vcftools, bcftools, bedtools among others. If working on a RedHat operating system, the package manager YUM (Yellowdog Updater, Modified) (https://access.redhat.com/solutions/9934) is the equivalent of APT.

**Conda**

Conda (https://docs.conda.io/en/latest/) is also a package management tool, though it sits somewhere between package managers like APT and containers (see below) due to its ability to also manage environments (i.e., collections of software). This feature makes conda extremely useful, particularly for bioinformatics software where different pipelines may utilise the same tools but require different versions of a particular tool. Conda allows you to easily install and run pipelines in their own separate environments so they do not interfere with one another and also enables you to easily update software when new versions are made available. Bioconda [22] is a channel for conda which specialises in bioinformatics software and includes a myriad of the most commonly used bioinformatic tools. Furthermore, conda also enables the installation and management of popular programming languages such as python or R, along with their respective libraries and packages. It is a great resource for bioinformaticians of all levels and is particularly helpful as a stepping-stone before stepping down a container lane.

**Containers**

Containers package up software and all dependencies, as well as all of the base system tools and system libraries into a separate environment so that they can be reliably run on different computing platforms. Containers are similar to conda environments, but they differ in the sense that containers include absolutely everything they need within the container itself (even including the base operating system). It is sometimes easier to think about containers as installing a whole separate machine that just utilises the same computing resources and hardware as the local machine it is installed on. The main advantage of a container over a conda environment is the ease of reproducibility due to the ability to pull a specific container each time you want to run, or re-run, a certain pipeline or use a particular tool, no matter what computing platform you are using. Reproducibility can be achieved with conda environments too, but this often requires exporting and keeping track of saved environments.

There are 2 main options when wanting to use a container: Docker [23] or Singularity [24]. Docker is the most standard container service available with thousands of containers available from DockerHub (https://hub.docker.com) or from other container registries such as quay.io (https://quay.io). Bioinformatics software that is available via bioconda also has a respective docker container on quay.io through the BioContainers architecture [25]. This means many common bioinformatics software and pipelines are already available in a containerised environment. Otherwise, some software developers make their own containers available, e.g., Trinity (for RNA-seq assembly) (see https://github.com/trinityrnaseq/trinityrnaseq/wiki/Trinity-in-Docker) or BUSCO v4 (for assessing assembly completeness) (see https://busco.ezlab.org/busco_userguide.html#docker-image). There are also thousands of other public docker containers across a range of online container registries that may have the software you are looking for, or there is always the option to create your own Docker container for reproducible pipelines. Obviously, Docker can be used to download and employ Docker containers, but Singularity is another program that can also be used to download and employ Docker containers (particularly on HPC environments). Both have advantages and disadvantages, so it is usually down to user preference as to which to choose. If you are new to containers, we suggest starting with Singularity. Not only will this allow you to easily be able to scale up your containerised pipelines to HPC environments but also makes reading and writing files to and from the container from the local machine a bit more straightforward.

**Manual installation**

If none of the above methods are available for your chosen software, you may need to install it manually. This process is usually explained step-by-step in the software documentation but typically involves a number of steps including: (1) Downloading a tar package (or zip file) of the source code (or cloning a Git repository) from GitHub (https://github.com) (or another website); (2) Unpacking the source code to extract its contents; (3) Configuring the software to check your environment and ensure all of the required dependencies are available; (4) Building the finished software from the source code; and (5) Installing the software, i.e., copying the software executables, libraries, and documentation to the required locations. This process is what package managers and containers do automatically for you. There are a number of standard dependencies that are usually required for manual installation (e.g., the build-essential package, the dh-autoreconf package, and the libarchive-dev package) so it is often handy to install these using APT before attempting to manually install any other software. You will be notified of any other required dependencies you may be missing during the installation process.

Once you have your software installed, it is good practice to try and run the program with the help command-line option (i.e., -h/—help/-help), or with no parameters, to ensure it has been installed correctly. If the help option displays some information about running the program and the different command-line options, it is usually a good sign that your software was installed successfully and is ready to go. If your tool does not seem to be working, you may need to ensure the executable for your tool (and sometimes its required dependencies) is available in your path. But what exactly is your path and why is it important? Well, whenever we call upon a particular input file or output directory within a command, we often use an absolute or relative path to show the program where that file or directory is sitting within the file system hierarchy. We can also call upon tools or executables the same way, though it is not efficient to provide a path to a tool every time we need to use it. The path environmental variable overcomes this issue by providing a list of directories that contain tools/executables you may wish to execute.

By default, the path variable is always set to include some standard directories that include a variety of system command-line utilities. So, to ensure a new program can be called upon anywhere without specifying the path to the program, you can either move or copy the tool/executable to a directory that is already listed in your path variable, or add a new directory to the path variable that contains the program. New directories can be added to your path either temporarily (by simply exporting the path variable with the added directory included) or permanently (by editing your.bash_profile). Another thing to be aware of is that the order of directories in your path is important because if the same program (or executable with the same name) is found in 2 different directories, the one that is found first in your path will be used. Always keep this in mind when adding new directories to your path to determine where they should sit in the list of paths. [The sheer number of times we mentioned the word “path” in this rule alone should emphasise how important paths really are—though we promise there are no more mentions of it for the rest of this article].

### Rule 6: Carefully curate and test your scripts

In other words, always double-check (or triple-check) your scripts and perform test runs at each step along the way. Before you run your pipeline, it is important to first read through the software documentation to ensure you understand the different inputs, outputs, and analysis options. Ensure that the documentation is for the correct version of the software as particular command-line options may change version to version. Many bioinformatics programs have extensive documentation online, either through their GitHub or another website. The basic documentation for most tools can be accessed using the command-line help options (which is also a great way to determine whether your required tool is available and installed correctly—see Rule 5). Sometimes more detailed information can be found in a README file in the source code directory. Most documentation should provide some example commands on how to run the program with basic or default options which should assist you in curating a successful script.

Once you have your final script, it is essential to give it a quick test to determine if there are any immediate errors that will prevent your script from running successfully. From simple spelling mistakes or syntax errors which result in files or directories not being found or commands being confused with invalid options, to not being able to locate the desired software or the software being configured incorrectly with problematic dependencies. These are the “face-palm” errors that any bioinformatician is aware of as we have all been there, time and time again. The good news is that these errors are often quite simple to fix. Yet it is better to catch them early rather than waiting in queues only for your script to error as soon as it starts, or leaving your script to run in the cloud only to come back and realise the machine has been sitting there idle the whole time due to a minor scripting error. Testing your scripts in the cloud is usually as simple as running the script or command and watching to see whether any errors are immediately thrown on-screen, but to test scripts in a shared HPC environment, you may need to utilise an interactive queue. Interactive queues allow you to run commands directly from the command line with a small subset of HPC resources. These resources are usually not enough to run an entire pipeline but are quite useful for testing and debugging purposes. Obviously, your script may still run into errors later on in your pipeline, but testing your script before you submit it properly should alert you to any preliminary errors that would prevent the pipeline from starting successfully and prevent any precious time being wasted in queues or precious dollars being wasted on idle cloud compute.

### Rule 7: Monitor and optimise your pipelines

Once you have your script running, it is important to monitor your pipelines to determine whether it is effectively utilising the computational resources you have allocated to it. Understanding what resources your pipeline utilises can help you scale up or down your compute so that you are not wasting resources or hitting resource limits that may slow down your pipeline. On shared HPC infrastructure, you will usually be able to see a summary of the computational resources used from either the job log files or scheduler-specific commands. Metrics such as maximum RAM and CPU usage as well as CPU time and walltime are useful in adjusting future scripts so that they request the optimum amount of resources needed. This enables the pipeline to run efficiently without any unnecessary queue time. Storage space of output files should also be monitored periodically to ensure you are not exceeding your allocated quota.

More specific monitoring is possible when running pipelines in the cloud as you have full control over all computing resources. Simple programs like htop (https://hisham.hm/htop/) can be used for fast real-time monitoring of basic metrics like CPU and RAM usage, while more in-depth programs like Netdata (https://www.netdata.cloud) can assist with tracking a large variety of metrics both in real-time and across an entire pipeline using hundreds of preconfigured interactive graphs. Many bioinformatic pipelines are “bursty” in nature, meaning different steps in a single pipeline may have vastly different computing requirements. Some steps/tools may have high memory requirements but only utilise a small number of cores, while others may multithread quite well across a large number of cores but require minimal memory. Knowing the required computing resources for each step may help you break up your pipeline and run each stage on a different machine type for greater cost efficiency. Monitoring disk space requirements throughout a pipeline is also important as many bioinformatics tools require large amounts of temporary storage that are often cleaned upon completion of the pipeline. Attached storage can be quite costly in the cloud, so ensuring you only request what is necessary will also reduce pipeline costs.

Overall, monitoring of bioinformatics pipelines is key to improving pipeline efficiency, optimising computing resources, reducing wasted queue time, and reducing cloud costs.

### Rule 8: Get familiar with basic bash commands

As a bioinformatician, your main role is to make sense of biological datasets, and this often means manipulating, sorting, and filtering input and output files to and from various bioinformatic tools and pipelines. For example, you may want to extract information for a certain sample or a certain gene of interest. Or in a file containing a table of data, you may want to sort an output file by a particular column or select rows that contain a particular value. You may want to replace a certain ID with a respective name from a list or perform a calculation on values within a column. Fortunately, many of the input and output files used in bioinformatics are regular text files, so these tasks can easily be achieved. One might think about using common spreadsheet applications such as Microsoft excel to perform these tasks; however, while this may suffice for small files, excel is not too fond of the sometimes millions of rows of data that are characteristic of a number of common bioinformatic files. This is where some standard unix shell command-line utilities come into play, namely the grep, AWK, and sed utilities.

Global regular expression print (grep) is a command-line utility which searches a text file for a regular expression (i.e., a pattern of text) and returns lines containing the matched expression (Table 1). This tool is useful when wanting to filter or subset a file based on the presence of a particular word or pattern of text (e.g., a sample name or genomic location, etc.). AWK is much more extensive command-line utility which enables more specific file manipulation of column-based files (Table 1). For example, AWK can return lines where a column contains a particular value or regular expression; in addition, it can output only particular columns, perform calculations on values within the columns, and work with multiple files at once. The extensive abilities of AWK are too grand to cover here but just know that this clever little tool will likely hold a special place in any bioinformatician’s heart. Lastly, stream editor (sed) has a basic “find and replace” usage allowing you to transform defined patterns in your text. In its most basic form, sed can replace a word with another given word (Table 1) but can also perform more useful functions like removing everything before or after a certain pattern or adding text at certain places in a file.

**Table 1:** Basic usage examples of the grep, awk, and sed commands.

Command	Example	Description
grep	grep "chr5" file	Print all lines that contain the string "chr5" in the named file
awk	awk '$1 == 5 {print $2, $3}' file	For rows in the named file where the value in column 1 is equal to 5, print columns 2 and 3
sed	sed 's/sample1/ID7037/g’ file	Replace all occurrences of "sample1" with "ID7037" in the named file and print the result

Of course, grep, AWK, and sed all have their limitations, and more extensive file manipulation may be better suited to a python or perl script (and there is already a great “Ten simple rules” article for biologists wanting to learn how to program [26]); but for simple processing, filtering, and manipulation of bioinformatics files, look no further than these 3 useful command-line utilities.

### Rule 9: Write it down!

A previous “Ten simple rules” article has highlighted the importance of keeping a laboratory notebook for computational biologists [27], and another covered some best practices around the documentation of scientific software [28]. Many components from these articles apply to our rule of writing it down and keeping helpful notes when getting started with command-line bioinformatics. The number of pipelines or analyses that can be run on a single set of biological data can sometimes be quite extensive and usually coincides with a lot of trial and error of different parameters, computing resources, and/or tools. Even those with a great memory will often look back at results at the time of publication and ponder “why did we use that tool?”, or “what parameters did we end up deciding on for that analysis?”. Keeping detailed notes can be a real lifesaver. Not only is it important to keep track of your different script files, and the required computing resources for each script, but also the accompanied notes about why you chose a particular tool and any troubleshooting you had to do to run the pipeline successfully. An easy-to-access document of all of your favourite commands and nifty pieces of code that may come in handy time and time again is also a must! Getting familiar with helpful code text editors like Visual Studio Code (https://code.visualstudio.com), or Atom (https://atom.io), as well as investing some time into learning helpful mark-up languages like Markdown will assist with keeping detailed, organised, and well-formatted scripts and documentation for the pipelines you are using. Exactly how you decide to keep your notes is completely up to you, but just ensure to keep everything well-organised, up-to-date, and backed up. Also, publishing your scripts as markdown files in supplementary material ensures the utility (and citability) of your work.

### Rule 10: Patience is key

The number 1 key (that we’ve saved until last) to being a successful bioinformatician is patience. A large proportion of your time will be spent troubleshooting software installation, computing errors, pipeline errors, scripting errors, or weird results. Some problems are simple to solve, while others may take quite some time. You will likely feel that with every step forward, there is just another hurdle to cross. Yet if you are patient and push through every error that is thrown your way, the euphoria of conquering a bioinformatics pipeline and turning a big lump of numeric data or As, Ts, Cs, and Gs into something biologically meaningful is well worth it. Also, as many past “Ten simple rules” articles in this field have addressed, do not be afraid to raise your hand and ask for help when you get stuck. Most of the time, someone before you has been in the exact same situation and encountered the same error or tackled a similar problem. Google will become your best friend and first port of call when things are not going as planned. And on the rare occasion where endless googling leads you nowhere, talk with your peers and reach out to the bioinformatic community; people are often more than happy to share their knowledge and put their problem-solving skills to the test.

## Conclusion

In the new era of whole genome sequencing, bioinformaticians are now more sought-after than ever before. Stepping into the world of command-line bioinformatics can be a steep learning curve but is a challenge well worth undertaking. We hope these 10 simple rules will give any aspiring bioinformatician a head start on their journey to unlocking the meaningful implications hidden within the depths of their biological datasets.
